# Dysregulated Metabolites Serve as Novel Biomarkers for Metabolic Diseases Caused by E-Cigarette Vaping and Cigarette Smoking

**DOI:** 10.3390/metabo11060345

**Published:** 2021-05-29

**Authors:** Qixin Wang, Xiangming Ji, Irfan Rahman

**Affiliations:** 1 Department of Environmental Medicine, School of Medicine and Dentistry, University of Rochester Medical Center, Rochester, NY 14642, USA; Qixin_Wang@URMC.Rochester.edu; 2 Department of Nutrition, Byrdine F. Lewis School of Nursing and Health Professions, Georgia State University, Atlanta, GA 30302, USA; xji4@gsu.edu

**Keywords:** metabolome, TCA, lipids, e-cigarette, cigarette, biomarkers

## Abstract

Metabolites are essential intermediate products in metabolism, and metabolism dysregulation indicates different types of diseases. Previous studies have shown that cigarette smoke dysregulated metabolites; however, limited information is available with electronic cigarette (e-cig) vaping. We hypothesized that e-cig vaping and cigarette smoking alters systemic metabolites, and we propose to understand the specific metabolic signature between e-cig users and cigarette smokers. Plasma from non-smoker controls, cigarette smokers, and e-cig users was collected, and metabolites were identified by UPLC-MS (ultra-performance liquid chromatography mass spectrometer). Nicotine degradation was activated by e-cig vaping and cigarette smoking with increased concentrations of cotinine, cotinine N-oxide, (S)-nicotine, and (R)-6-hydroxynicotine. Additionally, we found significantly decreased concentrations in metabolites associated with tricarboxylic acid (TCA) cycle pathways in e-cig users versus cigarette smokers, such as d-glucose, (2R,3S)-2,3-dimethylmalate, (R)-2-hydroxyglutarate, O-phosphoethanolamine, malathion, d-threo-isocitrate, malic acid, and 4-acetamidobutanoic acid. Cigarette smoking significant upregulated sphingolipid metabolites, such as d-sphingosine, ceramide, *N*-(octadecanoyl)-sphing-4-enine, *N*-(9Z-octadecenoyl)-sphing-4-enine, and *N*-[(13Z)-docosenoyl]-sphingosine, versus e-cig vaping. Overall, e-cig vaping dysregulated TCA cycle-related metabolites while cigarette smoking altered sphingolipid metabolites. Both e-cig and cigarette smoke increased nicotinic metabolites. Therefore, specific metabolic signatures altered by e-cig vaping and cigarette smoking could serve as potential systemic biomarkers for early pathogenesis of cardiopulmonary diseases.

## 1. Introduction

E-cigarette (e-cig) vaping has been increasing rapidly in the United States during recent decades since e-cig is considered a relatively safer alternative to help quit smoking [1]. The e-cig devices deliver aerosolized e-liquid with different concentrations of nicotine. The constituents from e-cig liquid are usually propylene glycol (PG) and vegetable glycerin (VG), which are generally recognized as safe (GRAS). Although PG and VG are GRAS, the aerosolized constituents have proven to be toxicants [2]. It has been known that e-cig delivers more nicotine than cigarette smoke [3,4]. Furthermore, we have shown that e-cig vapor contained various chemical constituents that can affect the downstream metabolism [5]. Cigarette smoke is known to contain thousands of toxic chemicals [6]. The chemicals generated from e-cig or cigarette smoking as xenobiotic chemicals in human organisms could dysregulate metabolomics profiles [7,8,9,10] and increase the risk of lung diseases, even lung cancers [11]. Commonly, cotinine is one of the significant metabolites during nicotine degradation, which has been used to identify the smoker or e-cig user [12]. We have shown circulating biomarkers are increased from e-cig users or cigarette smokers, predicting the risk of lung and heart diseases [13,14]. Our results found e-cig vaping is more associated with bioenergy synthesis (TCA cycle) than cigarette smoking, while cigarette smoking leads to upregulated sphingolipid pathways.

Bioenergy synthesis, including gluconeogenesis, glycolysis, and the TCA cycle, is one of the major metabolic reactions in mitochondrion for generating energy among all the organs/tissues. Previous studies reported that e-cig vaping and cigarette smoking inhibited bioenergy synthesis and induced mitochondrial dysfunction [15,16]. Mitochondrial metabolism alternation in lungs was followed by cigarette smoke exposure [15,16]; e-cig exposure induced mitochondrial oxidative stress and DNA damage [17,18]. Interestingly, a previous study explained that circulated PG would be metabolized into lactic acid in the liver and go through the TCA cycle [19]. However, no study is available to show the bioenergy synthesis-related circulating metabolites in e-cig users and cigarette smokers compared to healthy controls. 

Sphingolipids are lipids that contain sphingoid structures and major constituents of the plasma membrane [20,21]. Recent studies have shown that sphingolipid metabolites regulate pulmonary inflammatory responses, and they are essential mediators in lung cancer [20,22]. Cigarette smoke-induced accumulation of sphingolipid metabolites in the lungs is mediated with mitophagy, necroptosis, autophagy, and oxidative stress [22,23,24,25]. Interestingly, previous reports have described dysregulated plasma sphingolipids associated with lung cancer and chronic obstructive pulmonary disease (COPD) phenotypes [26,27]. In this study, we determined the dysregulation of sphingolipid metabolites in plasma from cigarette smokers or e-cig users.

We collected plasma from healthy controls/non-smokers/non-users, e-cig users, and cigarette smokers for metabolite analysis. Our results showed that metabolites related to nicotine degradation are dysregulated in the plasma from both e-cig users and cigarette smokers. TCA cycle-related metabolites showed alternation only in the plasma of e-cig users, while sphingolipid metabolites presented dysregulation only in cigarette smokers’ plasma. 

## 2. Results

### 2.1. Global Metabolic Profiling of Plasma from Healthy Controls, E-Cig Users, and Cigarette Smokers Analyzed by UPLC-MS

**Figure 1:**
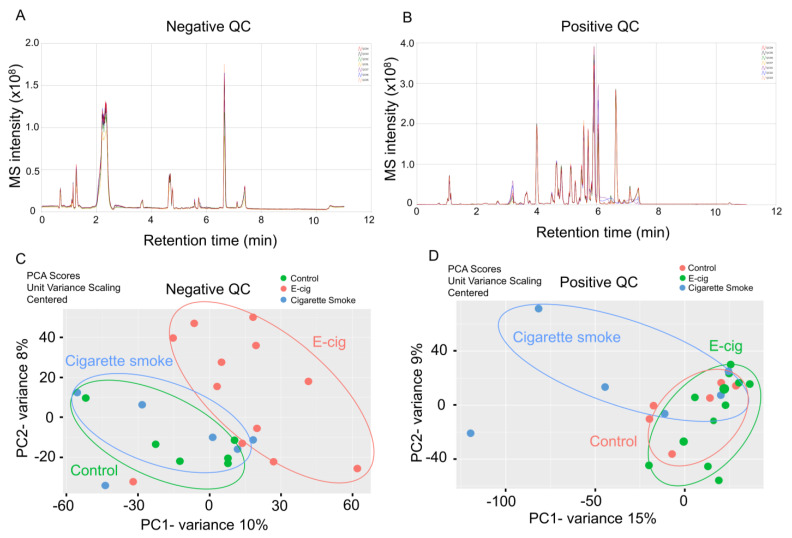
Metabolites from plasma were analyzed from ultra-performance liquid chromatography mass spectrometry (UPLC-MS). Spectra from UPLC-MS measured from (**A**) negative and (**B**) positive ion modes were used to identify individual metabolites. Score plots including all samples from principal component analysis (PCA) based on (**C**) negative and (**D**) positive ion modes presented dysregulated metabolomics affected by e-cig vaping and cigarette smoking.

We performed global metabolites profiling based on negative and positive ion modes to identify dysregulated metabolites in plasma from cigarette smokers and e-cig users through UPLC-MS (Figure 1A,B). We found a total of 1018 and 7244 metabolites that were detected in negative and positive ion modes, respectively. We further applied a multivariate analysis via the PCA model to determine the significance of metabolomics profiling in our cohorts (Figure 1C,D). In the negative ion mode UPLC-MS measurement, the absolute value of metabolites between the control and cigarette smoking groups are overlapped, while metabolites in the e-cig group show significantly different metabolites distribution (Figure 1C). Interestingly, we found overlapped metabolite distribution in the control and e-cig users’ plasma in positive ion mode, and cigarette smokers showed a significant difference in dysregulated metabolites (Figure 1D). 

**Figure 2:**
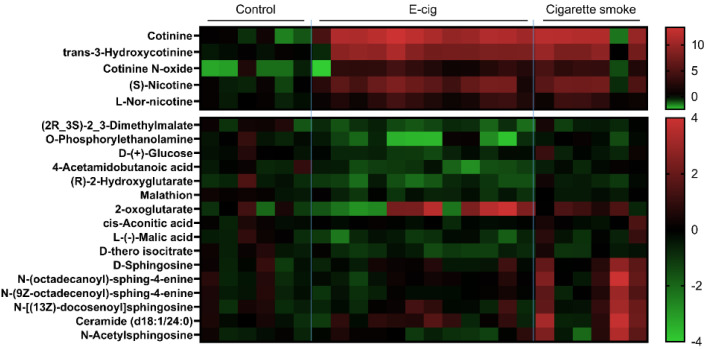
Metabolites from plasma were analyzed from UPLC-MS, metabolite fold changes were analyzed based on the normalized spectrum area. Heatmap representing significant dysregulated metabolites from nicotine degradation, TCA cycle, and sphingolipid metabolism among control (*n* = 6), e-cig (*n* = 12), and cigarette smoke (*n* = 6). Data are summarized as normalized log2 transformed.

We also screened and identified the dysregulated metabolic pathways in cigarette smoke and e-cig groups (Figure 2 and Table 1). Metabolic pathways, including nicotine degradation III, serotonin degradation, and gluconeogenesis, were altered in cigarette smoke and e-cig groups. Interestingly, the TCA cycle, d-galactose degradation, and UDP-N-acetyl-d-galactosamine biosynthesis II were found to be dysregulated in the e-cig group, while nicotine degradation IV was altered in the cigarette smoke group. Further, we identified specific dysregulated metabolites related to nicotine degradation, TCA cycle, and sphingolipid metabolism based on the normalized spectrum area (Figure 2). 

### 2.2. Nicotine Degradation-Related Metabolites Increased in Both Plasma from Cigarette Smokers and E-Cig Users

**Figure 3:**
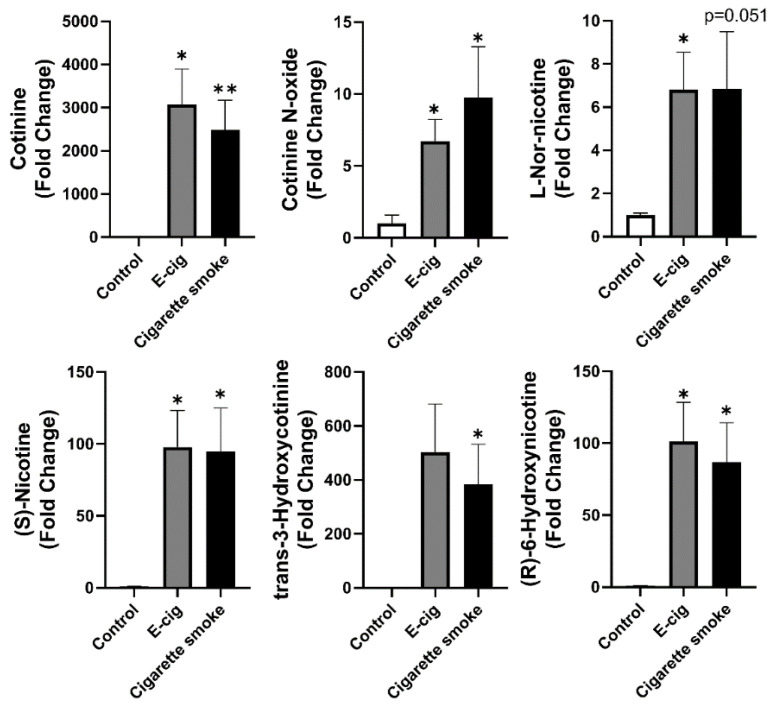
Metabolites from plasma analyzed from UPLC-MS from positive ion mode identified dysregulated nicotine degradation related metabolites in e-cig users and cigarette smokers. Fold changes were calculated based on the normalized area from UPLC-MS spectra, and control groups were used as a baseline. Data are shown as mean ± SEM (*n* = 6 for non-smoking control and cigarette smoke groups, *n* = 12 for e-cig group; * *p* < 0.05, ** *p* < 0.01 vs. control non-smokers).

Nicotine degradation is commonly seen after cigarette smoking and e-cig (with nicotine) vaping [28]. As expected, we have shown increased metabolites related to nicotine degradation in plasma from both e-cig users and cigarette smokers (Figure 3). Significantly increased metabolites were cotinine, cotinine N-oxide, l-nornicotine, (S)-nicotine, trans-3-hydroxycotinine, and (R)-6-hydroxynicotine (Figure 3). However, there was no significant difference between e-cig and cigarette smoke groups (Figure 3). l-Nornicotine, (R)-6-hydroxynicotine, and cotinine are the metabolic products converted from (S)-nicotine or nicotine; trans-3-hydroxycotinine and cotinine N-oxide are the downstream metabolites converted from cotinine.

### 2.3. Metabolites Associated with TCA Cycles Dysregulated in Plasma from E-Cig Users

**Figure 4:**
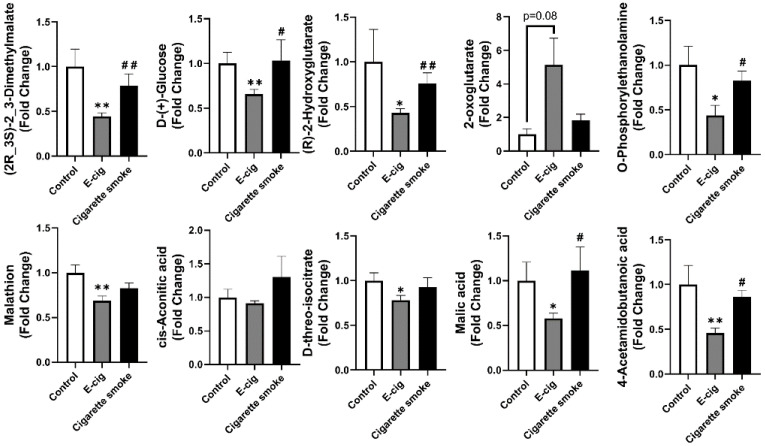
Metabolites from plasma analyzed from UPLC-MS from negative ion mode identified dysregulated TCA cycle related metabolites in e-cig users. Fold changes were calculated based on the normalized area from UPLC-MS spectra, and control groups were used as a baseline. Data are shown as mean ± SEM (*n* = 6 for non-smoking control and cigarette smoke groups, *n* = 12 for e-cig group; * *p* < 0.05, ** *p* < 0.01 vs. non-smoking control; # *p* < 0.05, ## *p* < 0.01 vs. e-cig).

Metabolites screened from the negative ion mode UPLC-MS were further identified, and we have shown that significant amounts of metabolites related to TCA cycles are statistically dysregulated in e-cig users’ plasma compared to cigarette smokers’ plasma (Figure 4). TCA cycle-related metabolites, such as (2R,3S)-2, 3-dimethylmalate, d-glucose, (R)-2-hydroxyglutarate ((R)-2-HG), O-phosphorylethanolamine, malathion, d-threo-isocitrate, malic acid, and 4-acetamidobutanoic acid (N-acetyl-GABA), are significantly decreased in e-cig users’ plasma compared to healthy controls or cigarette smokers (Figure 4). There was no change among groups for the concentration of cis-aconitic acid (Figure 4), and an increased plasma concentration of 2-oxoglutarate was found in e-cig users compared to control and cigarette smokers (Figure 4).

### 2.4. Dysregulated Sphingolipid Metabolites Found in Plasma from Cigarette Smokers

**Figure 5:**
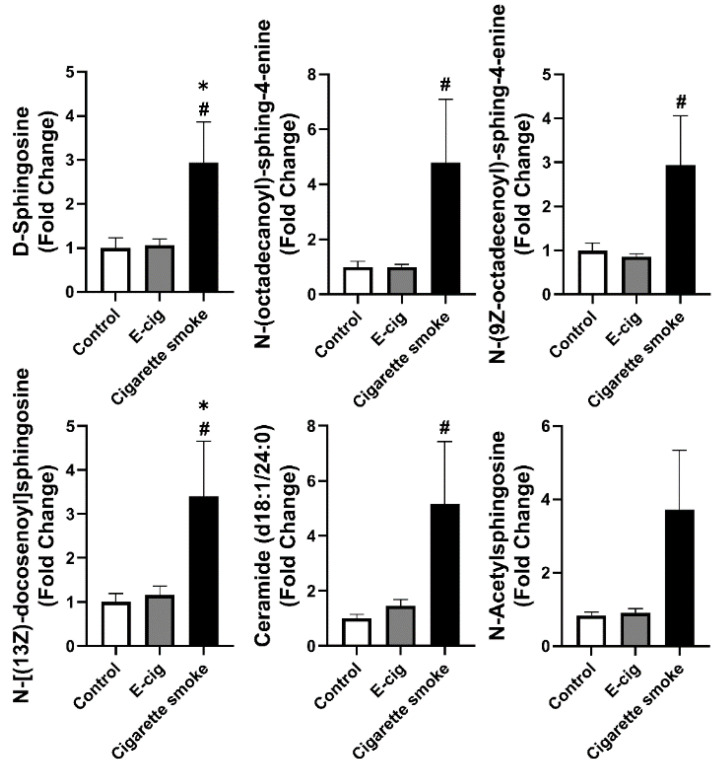
Metabolites from plasma analyzed from UPLC-MS from positive ion mode identified dysregulated sphingolipid metabolites in cigarette smokers. Fold changes were calculated based on the normalized area from UPLC-MS spectra, and control groups were used as a baseline. Data are shown as mean ± SEM (*n* = 6 for non-smoking control and cigarette smoke groups, *n* = 12 for e-cig group; * *p* < 0.05 vs. non-smoking control; # *p* < 0.05 vs. e-cig).

From the positive ion UPLC-MS analysis of screened metabolites, we have further shown that sphingolipid metabolites are dysregulated in cigarette smokers’ plasma compared to e-cig users or healthy controls (Figure 5). The concentrations of d-sphingosine, *N*-(octadecanoyl)-sphing-4-enine, *N*-(9Z-octadecenoyl)-sphing-4-enine, ceramide, and *N*-[(13Z)-docosenoyl]sphingosine were found to be significantly increased in plasma from cigarette smokers compared to e-cig users and healthy controls (Figure 5). The concentration of N-acetylsphingosine showed an increasing trend but not a significant difference between the cigarette smoke and control groups (Figure 5). 

### 2.5. Other Dysregulated Metabolites in Plasma Identified from E-Cig Users or Cigarette Smokers

**Figure 6:**
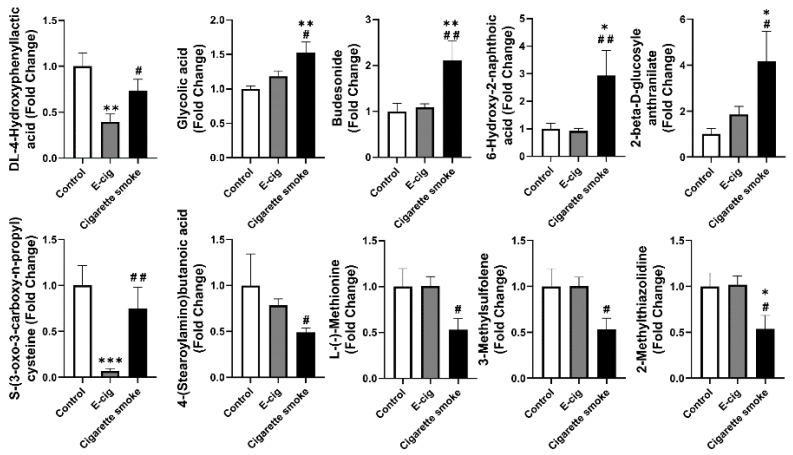
Metabolites from plasma analyzed from UPLC-MS from both negative and positive ion mode identified dysregulated metabolites in either e-cig users or cigarette smoker. Fold changes were calculated based on the normalized area from UPLC-MS spectra, and control groups were used as a baseline. Data are shown as mean ± SEM (*n* = 6 for non-smoking control and cigarette smoke groups, *n* = 12 for e-cig group; * *p* < 0.05, ** *p* < 0.01 vs. non-smoking control; # *p* < 0.05, ## *p* < 0.01 vs. e-cig).

In addition to the TCA cycle or sphingolipid metabolites, we also identified other significantly dysregulated metabolites (Figure 6). Among the metabolites significantly dysregulated in e-cig user’s plasma, we observed dl-4-hydroxyphenyllactic acid and S-(3-oxo-3-carboxy-n-propyl)cysteine were decreased in e-cig users compared to cigarette smokers and healthy controls (Figure 6). We found significantly increased metabolites such as glycolic acid, 6-hydroxy-2-naphthoic acid, 2-beta-d-glucosyle anthranilate, and budesonide, as well as significantly downregulated metabolites such as l-(−)-methionine, 2-methylthiazolidine, 4-(stearoylamino)butanoic acid, and 3-methylsulfolene (Figure 6). 

## 3. Discussion

E-cig vaping has rapidly increased since it has been presumed as a safe alternative to cigarette smoke, evoking public concerns about the health risks of e-cig vaping [29]. Our previous studies have shown that acute and chronic e-cig exposure can induce pulmonary inflammation and oxidative stress [30,31]. Many studies have shown that cigarette smoking induces metabolic disease with dysregulated metabolites [9,32]; however, limited studies have elucidated the effects of e-cig on metabolic disorders, which identify promising metabolite biomarkers related to potential diseases [32,33]. In this study, we have identified dysregulated metabolites from the plasma of e-cig users and cigarette smokers related to nicotine degradation, the TCA cycle, and sphingolipid metabolism as well as some other metabolites caused by e-cig vape aerosol and cigarette smoke. 

Nicotine degradation pathways are the most commonly activated metabolic responses in cigarette smokers and e-cig users (nicotine-contained in e-cig vaping). When nicotine from cigarettes and e-cig aerosols was inhaled into the human body, a number of metabolites are metabolized from nicotine [28]. The most important and commonly used metabolite to identify nicotine degradation is cotinine, which is converted from 70–80% of nicotine introduced into the human body [28]. The other cotinine-associated metabolites identified from our study, including cotinine N-oxide and trans-3-hydroxycotinine. Around 35~42% of the total cotinine will be transformed to cotinine N-oxide and trans-3-hydroxycotinine [28]. Nicotine-related metabolites, such as nornicotine and 6-hydroxynicotine, will be converted from nicotine [28]. About 10% of the nicotine will not be metabolized, and we have detected it as (S)-nicotine, and [28]. Cotinine has been used as a biomarker to identify nicotine degradation, which is the commonly activated metabolism after smoking and nicotine vaping [30,34]. Furthermore, nicotine, cotinine, cotinine N-oxide, and trans-3-hydroxycotinine are considered primary metabolites in total nicotine equivalent (TNE), which have been used as standards to validate nicotine intake [35]. Other metabolites such as nornicotine and 6-hydroxynicotine are less concentrated (< 2%) and lower in abundance than TNE metabolites [28]. Hence, they are not considered as regular biomarkers for the characterization of nicotine inhalation [28,35,36]. A previous study has identified that nornicotine preserves a longer half-life than nicotine or cotinine [37], and nornicotine was highly relevant to TNE in smokers’ urine than health control [37]. Consistent with these data, our results confirm that although nornicotine or 6-hydroxynicotine are low abundances in body fluids, they are still sufficient to serve as biomarkers to identify the smoking status and an indicator for nicotine degradation pathway activation. 

The TCA cycle is a series of biochemical conversions with the generation of bioenergy, which usually occurs in mitochondrion with the products from glycolysis. A previous study has shown that PG or PG/VG inhibited glucose metabolism and ATP generation in airway epithelium [38]. The aerosolized PG/VG inhaled into lungs were unlikely deposited and accumulated in the bloodstream since the half-life for PG is ~4 h; PG will be converted to lactic acid via alcohol dehydrogenase in the liver and then merged in the TCA cycle [19]. Our previous studies described that e-cig exposure can induce oxidative stress in the mitochondrion and dysregulation of mitochondrial complexes in lung fibroblasts [39]. Furthermore, e-cig exposure causes an increased amount of damaged mitochondrial DNA in plasma as well as increases the risk of cardiovascular diseases [18]. In this study, we showed that most of the TCA cycle-related metabolites are downregulated in e-cig users while there were no changes in the cigarette smokers compared to the healthy controls. A decreased (R)-2-HG level in e-cig users was found, and (R)-2HG has been proved to exhibit as an oncometabolite which is capable of inhibiting tumor growth [40]. E-cig vaping downregulated the level of (R)-2HG in plasma indirectly reveals the risk of carcinogenesis associated with vaping, and (R)-2HG can serve as a biomarker for identifying e-cig vaping and cancers [40]. We also found a lower plasma level of (2R,3S)-2,3-dimethylmalate in e-cig users compared to cigarette smokers and healthy controls, and (2R,3S)-2,3-dimethylmalate can serve as a precursor of pyruvate, which is a basic substrate for the TCA cycle. A decreased level of (2R,3S)-2,3-dimethylmalate is in line with the TCA cycle substrates we have discussed above, and it is a promising biomarker for reflecting e-cig vaping inducing inhibition of bioenergy synthesis and mitochondrial respiration.

This is the first study to report that various metabolites associated with the TCA cycle are altered in e-cig users, since former studies are focused on nicotine-related metabolites identified from e-cig users. Surprisingly, we did not find a significant difference between the cigarette smokers and the healthy controls about the TCA cycle metabolites in plasma. It is well known that cigarette smoke inhibits mitochondrial respiratory function and dysregulates the TCA cycle [15]. The dysregulated TCA cycle-related metabolites identified from the e-cig group provide information that vaping might associate with synthetic bioenergy metabolism. Therefore, a larger sample size is needed for future studies.

Sphingolipid metabolites are associated with lung inflammation, emphysema, and COPD [22,27]. Among all the known sphingolipid metabolites, sphingosine-1-phosphate (S1P) and ceramide are well-studied [20]. Increased ceramide levels found in the elastase-induced mouse emphysema model and ceramides inhibitors were capable of attenuating elastase caused airspace enlargement [41,42]. We found that the cigarette smoke group showed significantly higher plasma levels of ceramide and sphingosine than e-cig users and the healthy controls. Since chronic cigarette smoking is shown to cause COPD/emphysema, our results are indirectly in agreement with previous studies [41,42,43]. Additionally, ceramide accumulation and the disproportion of sphingolipids were identified from the lungs of COPD/emphysema patients and smokers [44]. We have observed increased sphingosine as well, which can be converted from S1P, which is one of the downstream products of ceramide. Both ceramide and S1P are involved in the pathogenesis of various lung diseases [22,45], which allows sphingosine to have great potentiality as a biomarker for lung disorders associated with cigarette smoke. Other sphingolipid metabolites, such as *N*-(octadecanoyl)-sphing-4-enine, *N*-(9Z-octadecenoyl)-sphing-4-enine, and *N*-[(13Z)-docosenoyl]sphingosine are also promising biomarkers for lung injury induced by cigarette smoke. 

Both cigarette smoking and e-cig vaping have been associated with increasing risks of cardiovascular diseases [46,47]. Nicotinic metabolites are the major metabolites after smoking and vaping. Nicotine has been identified to promote myocardial remodeling and fibrosis, increase the risk of sudden heart failure and tachycardia/ventricular fibrillation, and upregulate blood pressure [48]. Our results show increased nicotinic metabolites in e-cig users and cigarette smokers, emphasizing that both e-cig vaping and cigarette smoking increases the risks of cardiovascular diseases. Other than nicotinic metabolites, we have identified dysregulated TCA cycle-related metabolites, such as citric acid, l-malic acid, and N-acetyl-GABA, in e-cig users compared to control. Citric acid and l-malic acid have been proved with a protective effect on ischemic heart diseases [49]. N-acetyl-GABA is a metabolite of GABA that can regulate cardiovascular stress induced by hypertension, and a decreased GABA reflects the vulnerability of hypertensive heart disease [50]. Significant upregulation of sphingolipid metabolites in cigarette smokers was identified in this study, and a recent clinical report described serum sphingolipids as biomarkers of cardiovascular disease [51]. In our cohort, we found increased serum ceramide (d18:1/24:0) levels in cigarette smokers, and ceramide (d18:1/24:0) has been reported as a predictor for the risk of myocardial infarcts and stroke [52]. Our results emphasize that elevated sphingolipids in cigarette smokers could serve as biomarkers for cardiovascular diseases. 

We have also identified other metabolites dysregulated in either e-cig users or cigarette smokers. We found a decreased l-(−)-methionine in cigarette smokers’ plasma compared to e-cig users and healthy controls. A lower level of l-(−)-methionine was identified with increased metabolic rates, weight loss, and increased risk of acute myocardial infarction, which have also been shown in cigarette smokers [53,54,55]. Our results describe the possibility that dysregulated metabolites from cigarette smokers or e-cig users are associated with predicting the pathogenesis of cardiovascular diseases. 

Despite identifying specific metabolites associated with e-cig vaping and cigarette smoking, the other contributing factors other than e-cig or cigarette smoke possibly affect the results. From our study, we noticed neither sex- nor age-dependent manner alterations of metabolites. However, e-cig device brands, e-liquid ingredients, smoking/vaping duration, and smoking/vaping habits affect the specificity and accuracy of the outcomes. The e-liquid basic ingredients, such as humectants present in commercially available products, are similar to each other, and no significant difference was noticed in puffing topography throughout different studies [56,57]. Hence, we do not expect significant contributors to dysregulated metabolites except specifically for vaping and smoking per se. However, a larger sample volume is required to minimize the random errors introduced by other confounders, and more detailed subject screening criteria should be standardized to understand the role of other contributing factors to metabolomics. We have matched the mass spectrum against with our local database to ensure the features of identified metabolites, and cross-comparison with the online database could also be of benefit by improving our results and minimizing the false-positive identifications [58].

In conclusion, various dysregulated metabolites were identified from e-cig users and cigarette smokers compared to healthy controls/non-smokers. Dysregulated metabolites from both e-cig users and cigarette smokers were correlated with nicotine degradation. Dysregulated metabolites related to the TCA cycle were found only in e-cig users, and altered sphingolipid metabolites were shown only in cigarette smokers; specific dysregulated metabolites identified in different groups serve as novel biomarkers for vaping and smoking associated with metabolic diseases. Further biochemical measurements of altered metabolites are required to confirm our findings in a larger cohort.

## 4. Methods and Materials

### 4.1. Human Subjects

Participants in this study have provided information including age, sex, gender, and ethnicity. Detailed information about cigarette smoking, e-cig vaping, and health control allowed us to categorize the condition groups as described previously [59]. In brief, we established the following criteria to screen potential participants in different categories: (1) ages in between 21 and 65 years old; (2) healthy control subjects are defined as never having used tobacco products (cigarette smoke, waterpipe smoke, and cigar) or any e-cig products; (3) e-cig users as defined as never having used any tobacco products; (4) there is no history of chronic diseases in any of our participants; (5) there are no current respiratory infections or any anti-inflammatory/corticosteroid drugs in use in any of our subjects; (6) female participants are not currently breastfeeding or pregnant. Written informed consents were required from all participants, and individual subject information (age, sex, and ethnicity) was obtained through a questionnaire and verbal communication (Table 2).

### 4.2. Plasma Samples Collection

Blood samples were spin down at room temperature for 5 min, 1000 rpm, then transfer the plasma into a new tube and stored at −80 °C [60] for UPLC-MS analysis.

### 4.3. Chemicals

Methanol (LC-MS-grade, Fisher Scientific Inc, Pittsburgh, PA, USA), isopropanol (LC-MS-grade, Fisher Scientific Inc, Pittsburgh, PA, USA), acetonitrile (LC-MS-grade, Fisher Scientific Inc, Pittsburgh, PA, USA), water (LC-MS-grade, Fisher Scientific Inc, Pittsburgh, PA, USA), formic acid (99.5+%) (LC-MS-grade, Fisher Scientific Inc, Pittsburgh, PA, USA), ammonium acetate (LC-MS-grade, Fisher Scientific Inc, Pittsburgh, PA, USA), and ammonium hydroxide (LC-MS-grade, Fisher Scientific Inc, Pittsburgh, PA, USA) were used for preparation of mobile phases and solutions.

### 4.4. UPLC-MS Analysis

UPLC-MS analyses were performed at the Mass Spectrometry Core Facility at Georgia Institute of Technology according to previously described protocols [60]. 

In brief, samples were separated through chromatography with Ultimate 3000 UPLC (Thermo Fisher Scientific, Inc., Waltham, MA, USA) system with a Waters ACQUITY UPLC BEH C18, 2.1 × 50 mm, 1.7 μm particle column as reverse phase (RP) separation with water/acetonitrile (40:60 *v*/*v*) as mobile phase A, and acetonitrile/2-propanol (10:90 *v*/*v*) as mobile phase B, and total 10 mM ammonium formate and 0.1% formic acid additives were used to elevate the metabolites identification efficiency by a Q-Exactive HF Orbitrap mass spectrometer (Thermo Fisher Scientific, Inc., Waltham, MA, USA) [60]. The mobile phases included. For, In parallel, samples were separated via hydrophilic interaction chromatography (HILIC) with Waters ACQUITY UPLC HILIC, C18, 2.1 × 50 mm, 1.7 μm particle column, and water/acetonitrile (95:5 *v*/*v*), 10 mM ammonium acetate, and 0.05% ammonium hydroxide as mobile phase A, and acetonitrile with 0.05% ammonium hydroxide were used for mobile phase B [60]. Metabolites identifications were based on the spectrum from mass spectrometer (Thermo Fisher Scientific, Inc., Waltham, MA, USA). During sample processing, column temperature maintained at 55 °C, while samples were kept at 5 °C with auto-injection with the volumes of 5 and 2 μL in RP and HILIC methods, respectively as described previously [60].

After sample separation and mass-spectrometer identification, we applied top five data-dependent acquisition (DDA) to collected MS/MS spectrum at stepped normalized collision energy (NCE) of 10, 30, and 50 V. A parallel reaction monitoring (PRM) processing, were carried out at NCE from 10 to 40 V to reveal complete metabolites profiling [60].

### 4.5. Data Processing

Compound Discoverer v2.1 (Thermo Fisher Scientific, Inc., Waltham, MA, USA) and XCMS software were both used to identified the exact metabolites [60]. In brief, we applied the software to analyze our raw data set, and chromatographic alignment was performed, then peak picking and peak area integration were processed for metabolites quantification, followed by QC-based compound area normalization to quantify metabolite dysregulations [60]. Retention times < 0.5 min (Reverse phase) and < 0.9 min (HILIC) were not considered as unreliable identification. The screening criteria for differential metabolic indicators include *p* < 0.05, fold change > 2, or <0.5 as described [60]. Welch’s t-test with a Benjamini-Hochberg correction was applied to cigarette smoke vs. control, and E-cig user vs. control, and corrected with a *p*-value < 0.05 was considered a significant difference. A further selection of dysregulated metabolic pathways in the above cohorts was based on overlap size (>6). The tentative ID configuration based on spectral (MS2) matching against the database was performed [60] to minimize false-positive identifications (Table 3). 

The fold change of specific metabolite was calculated based on the normalized area from positive or negative mode spectra. In brief, normalized areas from the control group were averaged and used as the baseline. The individual normalized area from different samples was divided by the averaged normalized area from the control group as fold change compared to the baseline. 

### 4.6. Statistical Analysis

One-way ANOVA and Student’s *t*-test were used here to determine the significant difference in the change fold of metabolites among groups through GraphPad Prism Software version 8.0 (La Jolla, San Diego, CA, USA). Data were presented as mean ± SEM, and *p* < 0.05 was considered as a statistical difference.

## Figures and Tables

**Table 1:** Metabolic pathway dysregulation among non-smokers, e-cig users, and cigarette smokers.

Control vs. E-Cigarette				Control vs. Cigarette Smoke			
Pathways	Overlap Size	Pathway Size	*p*-Value	Pathways	Overlap Size	Pathway Size	*p*-Value
Nicotine degradation III	7	17	0.00361	Nicotine degradation III	6	17	0.00042
Serotonin degradation	5	7	0.00106	Serotonin degradation	3	7	0.00106
Gluconeogenesis	5	9	0.00138	Gluconeogenesis	4	9	0.00094
TCA cycle	8	9	0.00086	nicotine degradation IV	4	15	0.0016
d-galactose degradation V	6	6	0.00087				
UDP-N-acetyl-d-galactosamine biosynthesis II	6	7	0.00088				

**Table 2:** Patient information for subjects.

Group	Non-Smokers	E-Cigarette Users	Cigarette Smokers
Age	43.17 ± 7.00	40.50 ± 4.24	44.00 ± 4.59
Sex (Male/Female)	3/3	6/6	3/3
Ethnicity			
Caucasian/White	66.67%	41.67%	83.33%
African American	16.67%	25.00%	16.67%
Asian	16.67%	8.33%	0
N/A	0.00%	25.00%	0

**Table 3:** Spectral (MS2) matching description of dysregulated metabolites.

Name	Confidence Level *	Neutral Elemental Formula	Average Neutral MW	Average RT	Ion Type Detected	Theoretical Neutral Mass	Mass Error (ppm)	Description
Cotinine	2	C_10_H_12_N_2_O	176.0950	2.70	[M+H]^+^	176.0950	0.0	MS2 matched to MZCloud
Cotinine N-oxide	2	C_10_H_12_N_2_O_2_	192.0899	4.45	[M+H]^+^	192.0899	0.0	MS2 matched to MZCloud
l-Nornicotine	3	C_9_H_12_N_2_	148.1001	4.53	[M+H]^+^	148.1000	0.7	Two RT 4.530 and 5.374
(S)-Nicotine	2	C_10_H_14_N_2_	162.1157	5.47	[M+H]^+^	162.1157	0.0	MS2 matched to MZCloud
trans-3-Hydroxycotinine	2	C_10_H_12_N_2_O_2_	192.0899	2.65	[M+H]^+^	192.0899	0.0	MS2 matched to MZCloud
(R)-6-Hydroxynicotine	3	C_10_H_14_N_2_O	178.1106	6.00	[M+H]^+^	178.1106	0.0	MS2 matched to MZCloud
(2R,3S)-2,3-Dimethylmalate	3	C_6_H_10_O_5_	162.0526	1.35	[M-H]^−^	162.0528	−1.2	No MS/MS
d-(+)-Glucose	2	C_6_H_12_O_6_·H_2_CO_2_	226.0688	3.27	[M+HCO2]^−^	226.0689	−0.4	MS2 matched at 226.0689 M+H_2_CO_2_
(R)-2-Hydroxyglutarate	3	C_5_H_8_O_5_	148.0371	1.41	[M-H]^−^	148.0372	−0.7	MS2 matched to MZCloud (two RT 1.409 and 2.769)
2-Oxoglutarate	2	C_5_H_6_O_5_	146.0214	1.98	[M-H]^−^	146.0215	−0.7	MS2 matched to MZCloud
O-Phosphorylethanolamine	2	C_2_H_8_NO_4_P	141.0190	6.47	[M-H]^−^	141.0191	−0.7	MS2 matched to MZCloud
Malathion	3	C_10_H_19_O_6_PS_2_	330.0361	7.44	[M-H]^−^	330.0361	0.0	malathion is a man-made insecticide
cis-Aconitic acid	2	C_6_H_6_O_6_	174.0162	0.84	[M-H]^−^	174.0164	−1.1	MS2 matched to MZCloud (3 RT 0.843, 1.195, 2.169)
d-threo-Isocitrate	4	C_6_H_8_O_7_	192.02687	6.72	[M-H]^−^	192.0270	-	8 peaks 5.387–7.169
Malic acid	2	C_4_H_6_O_5_	134.0216	1.36	[M-H]^−^	134.0215	0.7	MS2 matched to MZCloud
4-Acetamidobutanoic acid	2	C_6_H_11_NO_3_	145.0739	1.56	[M+H]^+^	145.0739	0.0	MS2 matched to MZCloud
d-Sphingosine	2	C_18_H_37_NO_2_	299.2826	3.84	[M+H]^+^	299.2824	0.7	MS2 matched to local Database and MzCloud
*N*-(Octadecanoyl)-sphing-4-enine	3	C_36_H_71_NO_3_	565.5437	1.12	[M+H]^+^	565.5434	0.5	No MS/MS
*N*-(9Z-Octadecenoyl)-sphing-4-enine	3	C_36_H_69_NO_3_	563.5281	1.12	[M+H]^+^	563.5277	0.7	No MS/MS
[SP(20:0)]*N*-(Eicosanoyl)-sphing-4-enine	3	C_38_H_75_NO_3_	593.575	1.12	[M+H]^+^	593.5747	0.5	No MS/MS
[SP(22:0)]*N*-(Docosanoyl)-sphing-4-enine	3	C_40_H_79_NO_3_	621.6063	1.12	[M+H]^+^	621.6060	0.5	No MS/MS
*N*-[(13z)-Docosenoyl]sphingosine	3	C_40_H_77_NO_3_	619.5906	1.11	[M+H]^+^	619.5903	0.5	No MS/MS
Ceramide (d18:1/24:0)	2	C_42_H_83_NO_3_	649.6376	1.12	[M+H]^+^	649.6373	0.5	MS2 matched to local Database and MzCloud
dl-4-Hydroxyphenyllactic acid	2	C_9_H_10_O_4_	182.0579	1.188	[M-H]^−^	182.0579	0.0	MS2 matched to local Database and MzCloud
S-(3-oxo-3-Carboxy-n-propyl) cysteine	3	C_7_H_11_NO_5_S	221.0361	1.245	[M-H]^−^	221.0358	1.4	MS2 does not match well to in silico prediction
Glycolic acid	3	C_2_H_4_O_3_	76.0160	1.368	[M-H]^−^	76.0160	0.0	
2-beta-d-Glucosyle anthranilate	3	C_13_H_17_NO_7_	299.1007	6.46	[M+H]^+^	299.1005	0.7	This structure has the amine group ortho, and there is also an isomer where amine is para.
4-(Stearoylamino)butanoic acid	4	C_22_H_43_NO_3_	369.3246	1.082	[M+H]^+^	-	-	MS2 does not match well to in silico prediction
l-(−)-Methionine	2	C_5_H_11_NO_2_S	149.0511	4.855	[M+H]^+^	149.0511	0.0	MS2 matched to MZCloud
3-Methylsulfolene	4	C_5_H_8_O_2_S	132.0246	4.862	[M+H]^+^	-	-	MS2 does not match well to in silico prediction, isotopic pattern did not match sulfur-containing formula
2-Methylthiazolidine	3	C_4_H_9_NS	103.0456	4.861	[M+H]^+^	103.0456	0.0	MS2 matches in silico prediction, isotopic pattern suggests sulfur-containing formula

* Confidence Levels: (1) Match to authentic standard; (2) Match to MS/MS spectra in public database (MzCloud, ThermoFisher Scientific, Waltham, MA, USA); (3) Match to accurate mass in public database (Chemspider) or internal mass list; and (4) Unknown.

## Data Availability

The availability of all data presented in this study is from authors upon request.
